# Legros and Onimus upon the Influence of Electric Currents upon the Nervous System

**Published:** 1870-07

**Authors:** 


### Original Translations.

ARTICLE I. — *Legros and Onimus upon the Influence of Electric Currents upon the Nervous System.* From the Journal de l'Anatomie et de la Physiologie. (Robbins.)

[Continued from June No., 1870.]

§ 6. — *Derived Currents.*

We have stated how derived currents are formed, and how they were manifested in the organism. However relative to the direct current, their action is very feeble, and it may be said, in general, that their influence in man never occasions the disappearance of that of the direct current. It is not so, however, in experiments made upon animals.

Let us suppose a sciatic nerve traversed, during a long time, by a direct current, and that, at each closing of the current, we may have obtained a contraction, and nothing at the opening. We shall see, in proportion as the nerve loses its excitability, the phenomenon become inverse, that is, the contraction shall take place at the opening and not at the closing. The reasons for this phenomenon are as follows: A determinate portion of an electrized nerve being exhausted, the direct current no longer excites this portion, and, consequently, no longer determines contractions at the moment of its closure. But, at the same time that a direct current is established in the same portion of the nerve, a derivative current is formed, which is directed from the muscles of the thigh to the nerve. This current action of nervous terminations, and, consequently, upon nerves not exhausted, will determine an excita tion of these nervous filaments, and as it is ascending, the currents will take place at the opening, and not at the closing.

One might be astonished at the considerable action of derived currents in the organism, and we have stated the error which we may have committed, by considering as reflex the contractions provoked by derived currents. It is important to recall, here, that nerve, although a conductor of electricity, is, however, a bad conductor, and that explains to us why, when the resistance to the direct current is very strong, the derived currents assume an influ ence so considerable.

In order that the derived currents may thus change the order of the contractions, it is necessary, as we have said, that the portion of the nerve traversed by the direct current should be no longer excitable. Indeed, by irritating this portion of the nerve by mechanical or chemical excitants, contractions are no longer obtained from the appearance of the influence of derived currents.

MM. Longet and Matteucci have established the fact that, in electrizing mixed nerves, with a direct current, contractions were obtained at the closing, and with an inverse current at the opening; whilst in electrizing the anterior roots, that is to say, nerves purely motor, contractions took place at the commencement of the inverse current, and at the interruption of the direct.

MM. Martin Magron and Rousseau, whilst determining this fact, believe that they could explain it by derived currents. This objection is very serious, but we should add, that in the experiments of MM. Longet and Matteucci, it was necessary to take into consideration other conditions. Thus, M. Claude Bernard has observed, that the law of contractions is not the same, when the experiments are made upon mixed nerves separated from the nervous centre, or upon these same nerves intact and communicating with it. In this case, as in the experiments of MM. Longet and Matteucci, the contractions with an inverse current occur at the closing, and not at the opening.

If the facts observed by MM. Longet and Matteucci are not rigorously exact, in order to distinguish mixed nerves from nerves purely motor, it is then equally difficult to explain them by the single influence of derived currents.

It remains for us to speak of currents of polarization; these have the same cause as the secondary currents, only we distinguish them in this, that they occur only at the moment of the cessation of the current, whilst the secondary currents exist during all the time that the current traverses the tissues.

§ 7.—*Currents of Polarization.*

In the physical part, we have indicated the nature of currents of polarization. We have referred to experiments made upon animals both warm and cold blooded, and have arrived at this conclusion, that in an organic tissue traversed by a current, there is formed, at the moment of the cessation of the electrization, a direct current in an inverse direction. The experiments of Matteucci, which we have referred to in full, confirm this proposition, and give the explanation of these facts. Lastly, we have likewise instituted the following experiment, which is a new proof of this phenomenon: We immerse in two vessels, filled with distilled water, the extremities of a very sensitive galvanometer; then, by plunging the two hands into these vases, we observe, sometimes, that the needle remains at zero, at others, that it deviates slightly, either to the right or to the left. This deviation is always slight, but, nevertheless, it must be recognized.

Withdrawing, then, the hands from these two vessels, we plunge them into two others, which contain the poles of a continuous current apparatus. After having transmitted the current during a few seconds only, we replunge the hands into the vessels in contact with the galvanometer; immediately the needle is deflected thirty or forty degrees, or even more, and always in a direction opposite to the deflection which it would have experienced if it were influenced by the current used. It is possible, by always placing the same hands in the same vessels in communication with the galvanometer, to obtain a deflection in the opposite direction, if, the first time, the electrization is effected by a current going from right to left, and the second time by a current from left to right. The deflection of the galvanometer is, therefore, really the result of electric currents which continue after the electrization, and which occur in an inverse direction to the current employed. The deflection of the needle is so much the stronger, as the current has been more intense, or applied for a longer time. It occurs rapidly, and is not of long duration. It may be obtained uniformly if care is used to wipe the hands promptly before placing them in contact with the conducting wires of the galvanometer.

*There is formed, therefore, at the moment of opening the current, another current in the opposite direction to the primary current.*

This proposition will account for several phenomena which we have indicated in this chapter. We know, indeed, that when a current is transmitted through a galvanoscopic frog, there is obtained, at the opening of the circuit, a contraction only in the limb which has been traversed by the inverse current. This contraction is so much the stronger, as the passage of the current has continued longer, and as its intensity has been greater. Frequently this contraction becomes a true tetanic state.

Let us remember, that, with the weakest currents, it is the direct current which first determines contraction, which demonstrates that it is this which acts most energetically upon motor nerves. Now, when a nerve no longer produces contraction at the moment of the closure of an inverse current, but, on the contrary, produces it at the opening, it may be admitted that the contraction is due, not to the rupture of the inverse current, but to the action of the direct current of polarization, which is formed at the moment of the opening of the inverse current. This explanation, which was given for the first time by Matteucci, appears to be very exact, and we believe it applicable to the majority of cases.

On the other hand, whilst the motor nerve is influenced in the most energetic manner possible by the descending, the sensory nerve is excited especially by the ascending, current. If, then, a descending current be applied to a motor nerve, it is at the moment of the passage of the current that the nerve, stimulated to activity, will provoke muscular contractions. With an ascending current, as we have just seen, there is formed, at the opening, a descending current, and it is, in reality, at this moment that the contractions occur. Upon the sensitive nerve the ascending current will act at the moment of its application, and moreover, at the closing, reflex actions and evidences of pain are manifested; as to the descending current, it will not act, but at the moment of its opening, or at this instant there is produced an ascending current; and moreover, this is exactly what occurs, for we have already stated that the phenomena of pain are manifested especially at the cessation of the direct, and at the closure of the inverse, current.

The tetanic state sometimes apparent in the paw which has been traversed, during some minutes, by an inverse current, is likewise due to partial currents which are produced after the electrization. The proof of which is, that by cutting the nerve between the two poles, that is, by preventing the formation of secondary currents, this tetanic state never occurs.

The existence of these currents of polarization may be equally well established in man. We have, indeed, seen that by applying to the upper portion of the cord a sufficiently energetic ascending current, phosphenes appear, especially at the closure of the current, whilst, by employing a descending current, it is at the moment of the opening that the most intense phosphenes are produced. This fact seems explicable only by admitting, that at the moment of the rupture of the descending current an ascending current of polarization is formed, and as it is the ascending current which exercises the greatest influence upon reflex phenomena, it is this, probably, which is produced at the moment of the opening of the descending current, acts even as far as the brain, and likewise the optic nerve.

### § 8—*Difference of Excitability produced by Changes in the Intensity and Rapidity of the Current.*

Every change in the intensity of a current produced during the contraction of a muscle, is so much the stronger as the intensity of the current is greater. This proposition is only true up to a certain maximum, beyond which variations in intensity no longer produce any excitation. Upon this point we made the following experiment: through a frog, prepared after Galvani's method, the feeble current of Daniel's pile was passed, and whilst this current was maintained, if the influence of a second pile was added, contractions were still manifested at the closing. But if the current of two piles was passed in a continuous manner, the action of two, four, etc., other piles might even be added without exciting contractions: the limbs of the frog remain motionless. This may be readily conceived, for the excitation of the nerve has attained its maximum by the influence of the first two piles, and it can no longer be augmented by stronger currents. This fact demonstrates that in practice, when the excitation of a peripheral nerve is effected by a current of a certain intensity, it is useless to increase the energy of the current, for the effect would be no longer considerable, and there would be only the disadvantage of occasioning greater pain. In this same experiment, if, in place of transmitting the current in the same direction, we first employ a current from two piles, which is passed in a continuous manner from left to right, for example, and then transmit from right to left a current of equal force, or even two, three times weaker, a series of contractions will be effected, especially in the limb traversed by the ascending current. This fact demonstrates that a current, even much more feeble than another which already affects the nerves, occasions still further contractions when it is transmitted in an opposite direction, probably because it acts upon different nerves. The primary current being descending, for example, will act upon motor nerves, and an ascending current being applied during the passage of the first, to the same limb, will produce excitation of the sensory nerves, and may occasion reflex actions; the action of currents upon motor nerves is, therefore, to a great extent independent of that upon sensory nerves.

Every variation in the intensity of a current, produces in the nerve an excitation by as much stronger as the variation is more rapid (Dubois-Raymond). It is possible, then, by augmenting only with extreme slowness, to put in action upon the nerves a current somewhat intense and yet without producing contractions. To this end, it is necessary, without causing the slightest interruption, to add gradually the force of a new element. By this means the initial shock is avoided, which is always disagreeable, and which sometimes frightens the patient. This process is especially useful when it is desired to electrize the head or the cervical regions, for thus neither phospenes nor confusion will be occasioned. As at the opening of the current, a current is formed in an inverse direction, and just as strong as the current used has been intense; it is likewise necessary to diminish the current gradually; thus the shock of the rupture of the current and of the current of polarization will be avoided. It is possible, in this case, either to slide the reaphore, slowly, upon the adjoining parts when the epidermis is not moistened, which weakens the force of the current, or to diminish the number of the elements without causing any interruption at the moment when an element is removed; this result may be accomplished by means of a mechanical artifice which has been referred to in the continuous current apparatus.

In applications to the head, it is important never to arrest the electrization abruptly, for when this precaution is neglected, the patient experiences a shock, and quite a strong vertigo. The vertigo should especially be avoided, for it is impossible to be guided by what occurs during the moment of electrization, the vertigo in most cases appearing only at the moment of the rupture of the current.

### § 9.—*Different Opinions Expressed about the Influence of the Direction of the Currents.*

It would be impossible in this chapter to quote and to discuss all the opinions which have been expressed about the influence of electrical currents, when they act directly upon nerves; we shall investigate only the more recent, and those which have been sustained by authorities most justly celebrated. M. Claude Bernard, in his *Leçons sur la physiologie et la pathologie du systéme nerveux*, gives the following conclusions:*

"*a.* A nerve under its normal organic conditions, fitted to transmit voluntary excitations, when excited by galvanism makes but a single contraction, which occurs at the moment when the current is closed, whatever may be its direction.

"*b.* The nerve under these conditions, resembles muscle which never contracts but at the entrance of the current, whatever may be the direction of the latter.

"*c.* So that the actions of derived currents, so important to be considered on nerves separated from the individual, are without influence upon the nerve in its physiological state."

Upon a subsequent page, M. Claude Bernard, again adds the following conclusions, which are, so to speak, but the corollaries of the previous proposition:

"*d.* The phenomena which the nerve presents under the influence of an excitant, differ greatly, according as it may be contused, wounded, cut, or fresh, and in its normal relations.

* Page 185.

"*e.* All that has been said of the different directions in which contractions may be occasioned, does not apply to the healthy nerve, which always offers the same reactions, whatever may be the excitant to which it may be submitted, and whatever may be the mode in which this excitant be applied."

Our own experiments confirm many of the conclusions of M. Claude Bernard, and from the beginning we have had in view the proposition *d*, which is very important and which explains the majority of the contradictions occurring between different experimenters. We also reached this conclusion, that the action of derived currents is manifest only when the portion of the nerve traversed by the current is no longer excitable, which approximates closely to conclusion *c*, of M. Claude Bernard: "The actions of derived currents, so important to be considered on nerves separated from the individual, are without influence upon the nerve in its physiological state." Our conclusion differs from that of M. Claude Bernard in this, that we believe that, when the nerve is fresh and very excitable, the derived currents have no very manifest influence, whether the nerve be separated from the organism or not. Moreover, it is certain, as experiments made with the galvanometer prove, and as the phenomena of phosphenes and of metallic taste demonstrate, that derived currents exist in every case, and that they exert an influence more or less considerable.

As to the propositions *a*, *b*, and *c*, we believe them incomplete: for as we have already said, it is necessary to distinguish the cases in which nerves alone, from those in which nerves and muscles are acted upon. If in this last case, the contraction occurs especially at the moment of closing, it is not less true that the direction of the currents exhibits a very marked influence, and that under certain conditions there is a strong contraction at the opening of the current. We cannot animadvert sufficiently to this fact: that it is manifest that the direction of the currents has great influence upon the order and the energy of the contractions, and that this is important to be considered by the physiologist and the physician.

We shall perceive as we study pathological cases, how often this distinction is capital, and in a purely physiological point of view, we revert for these facts to paragraph (§ 1) one, and (§ 2) two, of this chapter, where we point out very marked differences, depending upon the directions of currents. This difference has been established since the time of Aldini and Volta, and we assume it as premise for the deduction of the laws which we have enunciated, not only from facts whichwe have observed, but, in addition, from the very exact and minute investigations of Matteucci.

Marianini (1), Matteucci (2), Remak (3), and several other authors, have asserted that the ascending current acts most energetically upon motor nerves, and the sensitive nerves are excited more strongly by the descending current, admitting, at the same time, that paralysis is a fact analogous to what we have been able to produce by constant currents of nerves having always the same direction; they have hence concluded that paralyses of motion should be treated by ascending, and paralyses of sensation by descending, currents. From the experiments which we have cited, it is, on the contrary, the opposite direction which should be preferred in each case.

Matteucci states, at the same time, that by means of constant currents, tetanic contractions may be arrested in a frog poisoned by strychnia, and that in this case supervenes without any contractions having taken place.

In order to accomplish the object of this experiment, says Matteucci, the ascending is preferable to the descending current, *the former producing less contractions at the beginning.*

This last phrase gives us the explanation of the erroneous ideas, in our opinion, of these different authors. It is true, and we have indicated it, that the ascending current determines in certain cases fewer contractions than the descending; but this difference is only accurate when the reflex actions are diminished or abolished. At the end of a certain time, in the case of tetanus, the ascending as well as the descending current, ought to induce an arrest of the tetanic contractions, for they both maintain the nerves permanently in the molecular state, which remaining constant can determine no muscular contraction. Excess of irritation can even produce this effect, but it seems to us dangerous to endeavor, as Nobili desired, to deaden the nerve in tetanus, it is better to act upon the sensitive nerves, and to make use of the current which in its order

(1) Marianini, Mémoire lu à l'Athénée de Venise. (*Annales de Chimie et de physique*, 1834.)

(2) *Traitè des phénomènes Electro-physiologiques des Animaux*, 1844, p. 270.

(3) *Galvanothèrapie.* Paris, 1866, p. 23.

prevents the action of sensitive nerves, and consequently causes a cessation of reflex contractions; now, this is the descending current.

In 1838, Matteucci, upon these theoretical ideas, attempted, with Doctor Farina, to cure a case of traumatic tetanus, by means of a constant current from a pile of thirty or forty elements. *An ascending current* was directed from the sacrum to the nucha. The tetanic contractions ceased, but the patient died nevertheless. Now, by this method of electrization, far from diminishing the excitability of the cord they increased it, and if the cure did not take place, it was due entirely to this permanent excitation of the cord, an excitation appears certainly to us, although the tetanic contractions had ceased.

We shall return to this subject when we come to discuss the action of electric currents upon reflex actions; but henceforth it is possible to judge the influence of physiological experiments upon therapeutices. It becomes, therefore, a matter of paramount importance to determine clearly, the physiological properties of different modes of electrization, and it is because we are persuaded of the truth of this proposition, we deem it useful to insist continually upon these facts, at the risk of being guilty of frequent repetition.

